# Supplementary material for: Ventricular and Atrial Remodeling after Transcatheter Edge-to-Edge Repair: A Pilot Study
Source: J Pers Med. 2022 Nov 16;12(11):1916. doi: 10.3390/jpm12111916 (PMC9696184; doi:10.3390/jpm12111916)
Supplement: Supplementary file 1 [file jpm-12-01916-s001.zip › jpm-1965710-supplementary.pdf]

Journal of Personalized Medicine

MDPI

Supplementary Materials

Ventricular and Atrial Remodeling after Transcatheter Edge-to-Edge Repair: A Pilot Study

Supplementary Table S1. Medical therapy before and after TEER procedure.

	Baseline (n=24)	Follow-Up (*n* = 24)	*p*-Value
Beta-blockers	21 (87.5)	21 (87.5)	1.000
ACE-I	4 (16.7)	4 (16.7)	1.000
ARB	8 (33.3)	10 (41.7)	0.766
MRA	22 (91.7)	22 (91.7)	1.000
Diuretics	23 (95.8)	23 (95.8)	1.000

Legend: ACE-I, angiotensin-converting enzyme inhibitors; ARB, angiotensin receptor blockers; MRA, mineralocorticoid receptor antagonist.

Supplementary Table S2. Baseline and follow-up assessment according to mitral regurgitation etiology.

	Baseline Assessment, Median [IQR]	Follow-Up Assessment, Median [IQR]	*p*-Value
	PRIMARY MR (*n* = 11)		
LVEDD (mm)	**50.0 [49.0-59.0]**	**46.0 [42.0–57.0]**	**0.005**
LVESD (mm)	42.0 [37.0-49.0]	40.0 [38.0–45.0]	0.284
RWT	0.32 [0.29-0.38]	0.35 [0.31–0.38]	0.424
iLVM (gr/m^2)	**111.1 [95.1-142.9]**	**79.2 [52.3–103.3]**	**0.005**
2D-iLVEDV (ml/m^2)	**61.4 [45.4-77.7]**	**48.9 [40.5-61.6]**	**0.003**
2D-iLVESV (ml/m^2)	25.2 [21.2-39.8]	20.4 [14.75–30.8]	0.182
2D-LVEF (%)	59.1 [50.7-61.5]	61.5 [50.0–63.9]	0.477
LV-S′ (TDI) (cm/s)	8.0 [7.5-9.5]	8.0 [6.0–10.0]	0.157
2D-iLAV (ml/m^2)	**42.3 [34.2-51.7]**	**36.3 [29.6–47.0]**	**0.005**
PALS (%) [N=3]	12.0 [4.0-17.0]	21.0 [19.0–22.0]	0.109
TR Vmax (m/s)	**3.0 [2.5-3.1]**	**2.0 [1.8–2.5]**	**0.005**
EPSPAP (mmHg)	**40.0 [35.0-50.0]**	**20.0 [15.0–25.0]**	**0.003**
Basal RVD (mm)	35.0 [31.0-40.0]	32.0 [29.0–37.0]	0.058
Mid-cavity RVD (mm)	**27.0 [25.0-34.0]**	**26.0 [23.0–31.0]**	**0.010**
Longitudinal RVD (mm)	**59.0 [52.0-68.0]**	**53.0 [47.0–60.0]**	**0.005**
RA Area (cm^2)	18.0 [15.0-23.0]	14.0 [13.0–16.0]	0.035
FAC (%)	**37.0 [35.0-44.0]**	**45.0 [43.0–47.0]**	**0.016**
TAPSE (mm)	20.0 [17.0-24.0]	20.0 [18.0-26.0]	0.072
RV S′ (TDI) (cm/s)	9.0 [8.0-13.7]	9.0 [8.7–13.0]	0.581
RV-FWLS (%)	-21.0 [-15.1—23.0]	- 23.0 [-24.0 – -22.0]	0.141
	Secondary MR (*n* = 13)		
LVEDD (mm)	60.0 [53.0-65.0]	60.0 [50.0–63.0]	0.058
LVESD (mm)	45.0 [40.5-52.0]	47.0 [41.5–50.0]	0.833
RWT	0.31 [0.26-0.37]	0.33 [0.25–0.38]	0.347

iLVM (gr/m^2)	116.9 [96.2-136.4]	100.8 [58.2–141.6]	0.239
2D-iLVEDV (ml/m^2)	**76.4 [60.5-94.9]**	**59.4 [44.8–91.2]**	**0.002**
2D-iLVESV (ml/m^2)	**50.2 [35.5-67.1]**	**31.9 [23.1–53.9]**	**0.004**
2D-LVEF (%)	42.4 [35.9-48.2]	46.3 [38.5–49.6]	0.433
LV-S′ (TDI) (cm/s)	6.5 [5.3-8.0]	8.0 [7.0–9.0]	0.105
2D-iLAV (ml/m^2)	53.8 [45.2-62.6]	50.7 [43.6–60.1]	0.213
PALS (%) [N=5]	10.0 [5.0-17.0]	6.8 [5.2–17.5]	0.500
TR Vmax (m/s)	2.9 [2.5-3.2]	2.4 [2.0–2.7]	0.056
EPSPAP (mmHg)	**42.5 [31.3-53.8]**	**30.0 [20.0–35.0]**	**0.005**
Basal RVD (mm)	**39.0 [37.0-43.0]**	**34.0 [32.0–38.5]**	**0.004**
Mid-cavity RVD (mm)	**30.0 [26.0-35.0]**	**28.0 [21.5–29.5]**	**0.016**
Longitudinal RVD (mm)	**65.0 [60.5-71.0]**	**60.0 [57.5–63.0]**	**0.012**
RA Area (cm^2)	**21.0 [17.5-25.0]**	**18.0 [15.5–22.5]**	**0.040**
FAC (%)	**34.0 [30.0-37.0]**	**42.0 [34.0–48.5]**	**0.009**
TAPSE (mm)	**14.0 [13.0-14.0]**	**17.0 [14.5–19.0]**	**0.005**
RV S′ (TDI) (cm/s)	7.0 [6.0-10.8]	9.5 [8.3–12.8]	0.068
RV-FWLS (%)	**-15.0 [-9.9 – 19.8]**	**- 19.5 [-16.0 – -22.5]**	**0.021**

Legend: EPSPAP, estimated peak systolic pulmonary artery pressure; FAC, fractional area change; iLAV, indexed left atrial volume; iLVM, indexed left ventricular mass; iLVEDV, indexed left ventricular end-diastolic volume; iLVESV, indexed left ventricular end-systolic volume; iRVEDV, indexed right ventricular end-diastolic volume; iRVESV, indexed right ventricular end-systolic volum; LAEF, left atrial ejection fraction; LVEDD, left ventricular end-diastolic diameter; LVESD, left ventricular end-systolic diameter; LVEF, left ventricular ejection fraction; LV S′, systolic velocity at the mitral annular; PALS, peak left atrial longitudinal strain; RA, right atrium; RVD, right ventricular diameter; RV-FWLS, right ventricle free wall longitudinal strain; RWT, relative wall thickness; TAPSE, tricuspid annular plane systolic excursion; TR, tricuspidal regurgitation.
